# Supplementary material for: Home Dust Mites Promote MUC5AC Hyper-Expression by Modulating the sNASP/TRAF6 Axis in the Airway Epithelium
Source: Int J Mol Sci. 2022 Aug 20;23(16):9405. doi: 10.3390/ijms23169405 (PMC9408837; doi:10.3390/ijms23169405)
Supplement: Supplementary file 1 [file ijms-23-09405-s001.zip › ijms-1801706-supplementary.pdf]

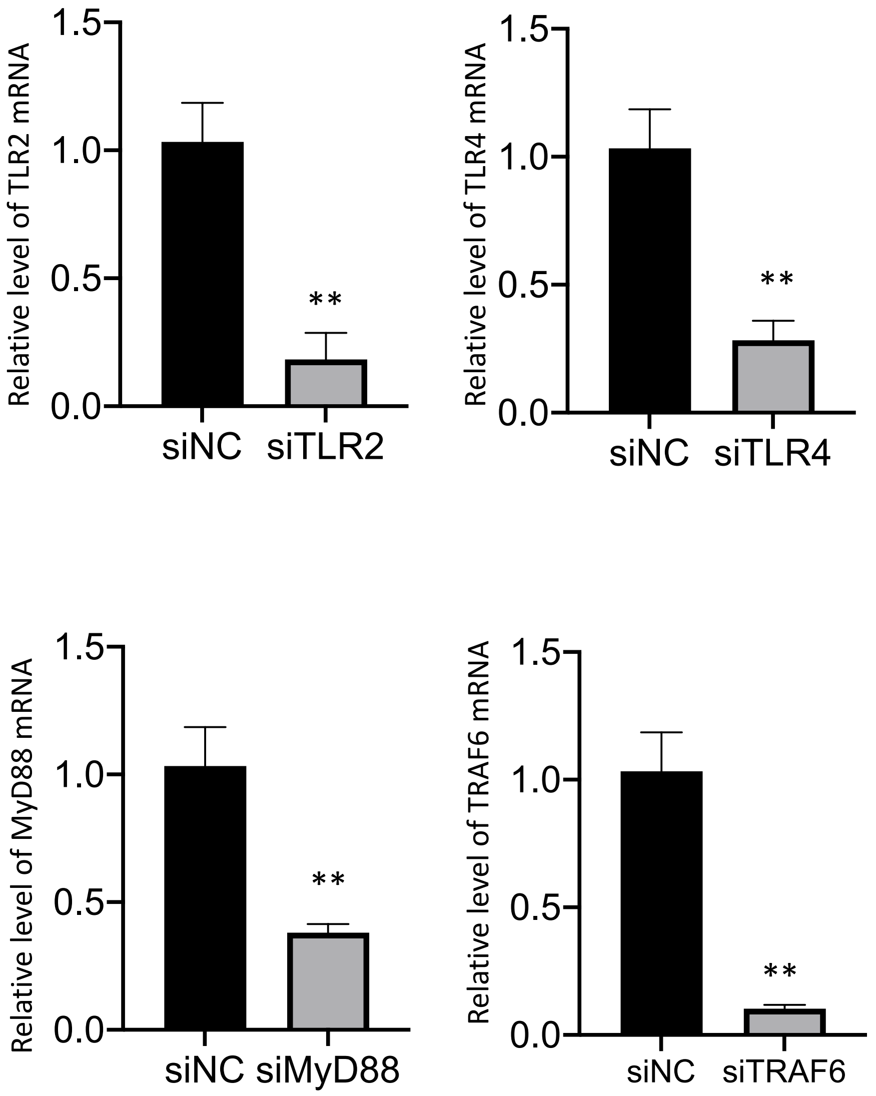

Figure S1. The siRNA knockdown efficacy in airway epithelial cells.
16HBE cells were transfected with indicated siRNA and results were normalized to the expression of ACTB (encoding β-actin) and control (siNC) cells. Data are representative of three independent experiments and presented as the mean ±SEM.
** $p < 0.01$

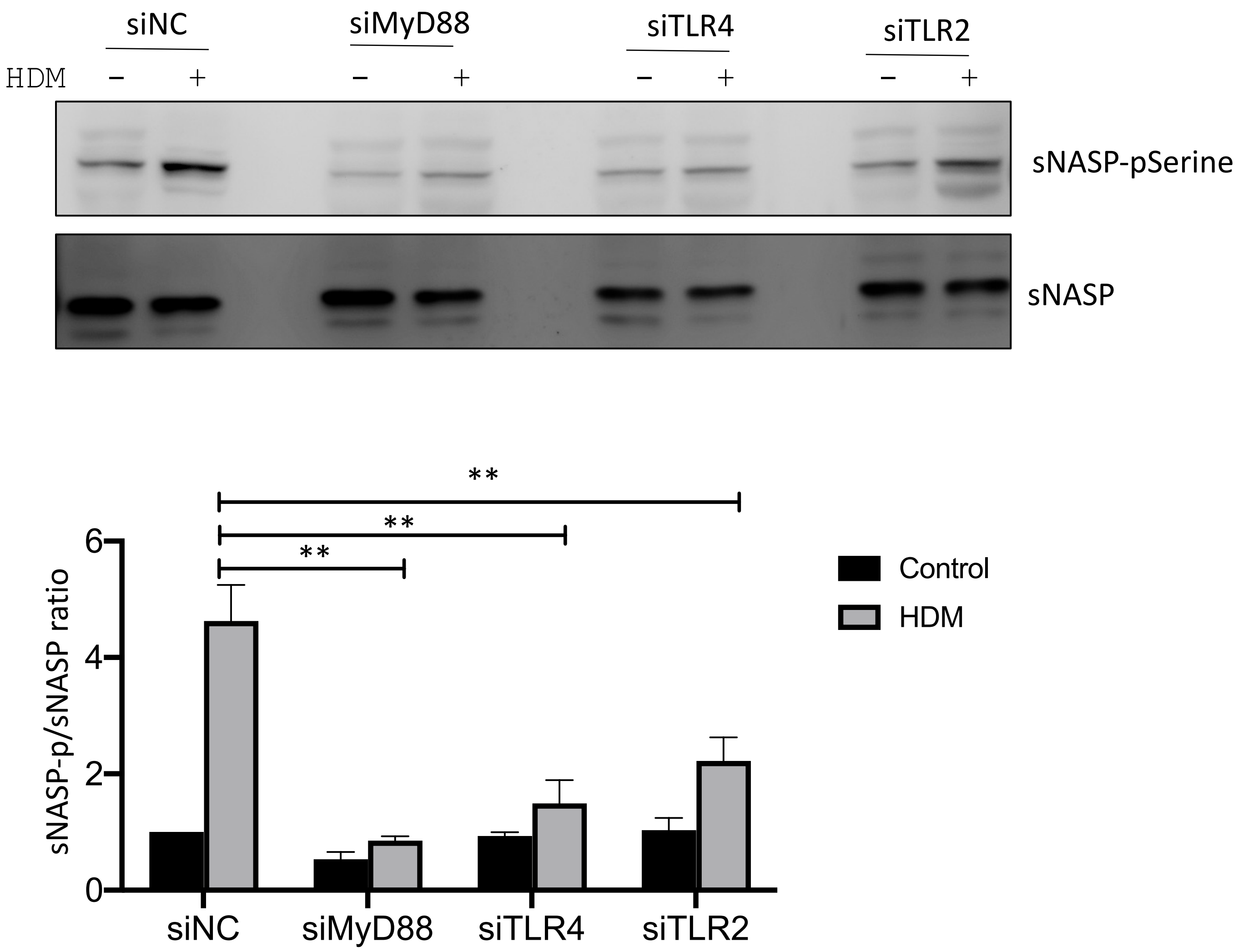

Figure S2. HDM mediates sNASP phosphorylation via TLR2/4-MyD88 signaling.
16HBE cells were transfected with indicated siRNA followed by stimulated with HDM for 24 h and assessed by an immunoblotting (IB) analysis with anti-pSerine158-sNASP and sNASP antibodies. Densitometric analysis of the Western blot shown below. Data are representative of three independent experiments and presented as the mean ±SEM. ** $p < 0.01$

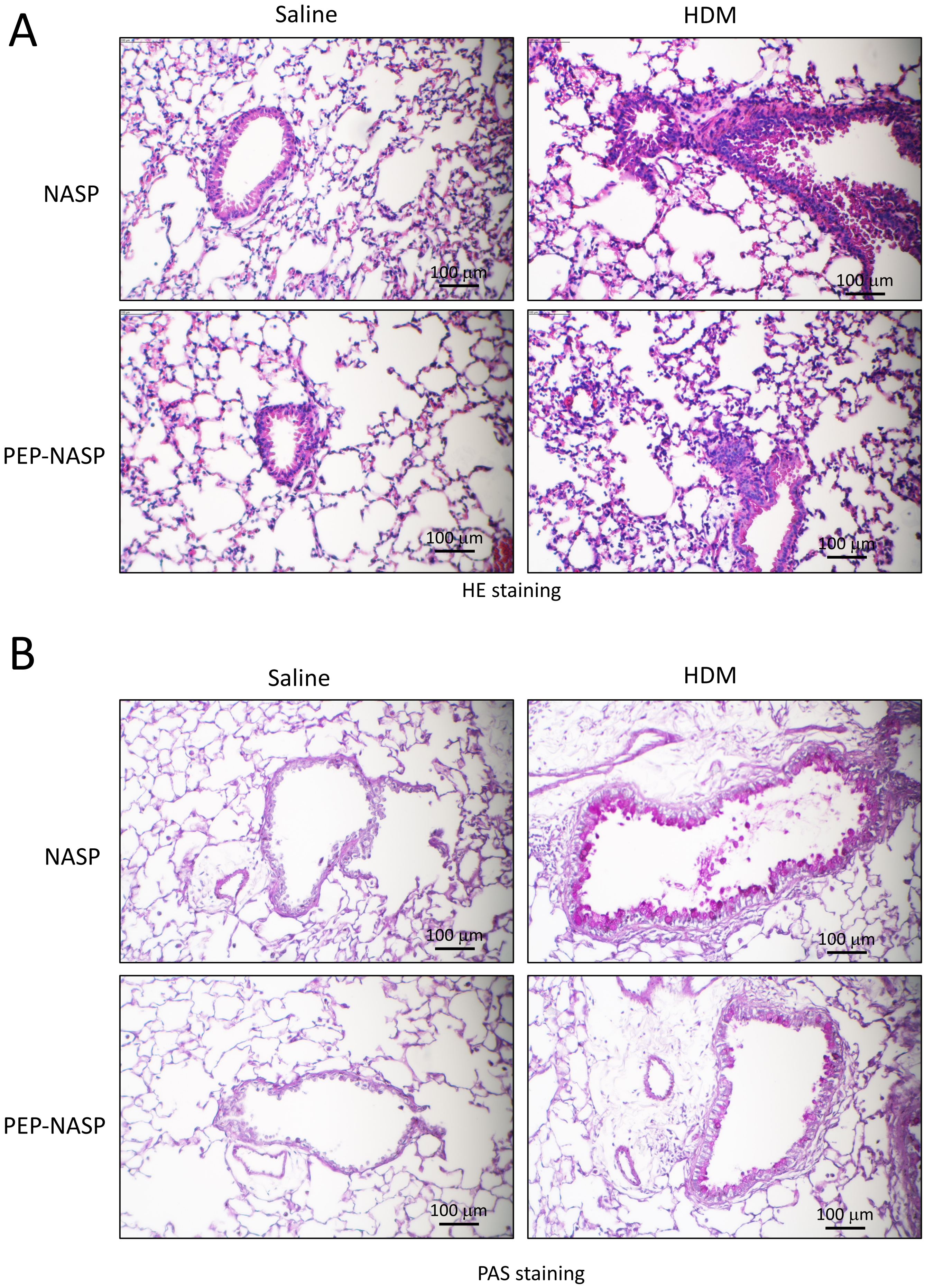

Figure S3. Effects of the PEP-NASP peptide on the inflammation and mucus production in airway.

Lungs from each group of mice were fixed and stained with H&E (A) and PAS (B). Images of lung sections were captured by microscope (magnification, x200); scale bar = 100 µm. (*n* = 10 per group per experiment).
